# Palliative Care in Children With Advanced Heart Disease in a Tertiary Care Environment: A Mini Review

**DOI:** 10.3389/fcvm.2022.863031

**Published:** 2022-04-08

**Authors:** Eva Bergsträsser, Saumya Lukose, Karin Zimmermann, Angela Oxenius

**Affiliations:** ^1^Pediatric Palliative Care, Department of Medicine I, University Children’s Hospital Zurich, Zurich, Switzerland; ^2^Department Public Health, Nursing Science, University of Basel, Basel, Switzerland; ^3^Pediatric Cardiology, Heart Center, University Children’s Hospital Zurich, Zurich, Switzerland

**Keywords:** congenital heart disease, advanced heart disease, pediatrics, palliative care, end-of-life care, pediatric cardiology, symptoms, suffering

## Abstract

Palliative care for children continues to evolve. More recently, this has also been true in the field of pediatric cardiology, particularly for children with advanced heart disease. In these children, similarly to children with cancer, treatment successes are offset by the risks of long-term morbidities, including premature death. This mini review aims to provide an overview of current knowledge on children suffering from advanced heart disease, their medical care during various phases of illness (including the palliative and end-of-life phase), symptom burden, experiences of parents, prognostic understanding of parents and physicians, and current status of the involvement of pediatric palliative care. In conclusion, the suffering of these children at the end of their young lives is pronounced and many parents feel prepared neither for medical problems nor for the child’s death. An effective and mutually trusting partnership between pediatric cardiology and pediatric palliative care would appear to be a prerequisite for the timely involvement of palliative care in further supporting these children and their families.

## Introduction

The outcome of children with severe congenital heart disease (CHD) has improved dramatically within the last decades. As a consequence, the population of children with a palliated and rather not repaired heart is growing. Many of these children suffer from a wide range of comorbidities, including associated syndromes. After extensive and repeated cardiac surgery for the underlying heart defect, many experience long-term morbidities, and an increasing number of children are referred to specialized pediatric palliative care (sPPC) services (1, 2). In these children, grouped as advanced heart disease (AHD) in this article, sPPC provides support that addresses the child’s and family’s most important needs and aims at improving the patient’s and family’s healthcare-related outcomes. This also includes processes of decision-making known as advance care planning (ACP).

The purpose of this mini review is to draw a picture of the current development and emerging concepts of sPPC in children with AHD and palliative care needs in the form of a summary of the current literature and an outlook on effective partnership between pediatric cardiology and sPPC in the context of tertiary care of well-resourced countries.

### Literature

#### Palliative Care in Children Suffering From Heart Disease

Unlike children suffering from, e.g., cancer, children with cardiac diseases have been rare in sPPC services. Consequently, the medical literature regarding palliative and end-of-life (EOL) care for these children is scant, starting only in 2010. One of the first retrospective studies delivered insight into EOL care and patterns of death for children with AHD (3). In addition to this article from the Children’s Hospital Boston, another article focusing on adults with complex CHD was published almost simultaneously and deserves attention (4). Tobler et al. (4) report a retrospective single center study including 48 patients (mean age 37 ± 14 years) with complex CHD who died in hospital unrelated to proximate surgery, two thirds of them in intensive care units (ICU). Circumstances of death, EOL discussions and EOL care are described, yet what strikes readers today is that only a minority of patients were informed and prepared for the terminal stage of their disease. Only 6% of the patients had EOL discussions, while 50% had full resuscitation status and died under full resuscitation efforts. However, as in the pediatric cohort (3), death was to be expected in these adult patients.

In the meantime, change has been emerging. A recent study from Boston compared two cohorts of inpatient pediatric deaths due to AHD from two three-year periods, from 2007 to 2009 and 2015 to 2018 (2). Of a total of 3409 cardiac admissions (2007–2009) and 4032 (2015–2018) in the two groups, 110 and 99 children died, respectively. sPPC involvement was documented more frequently in the later period, with 57 patients (58%) as compared to 19 (17%) in the earlier period.

#### Patterns and Medical Course of Congenital Heart Disease in Children

CHD is the most frequent birth defect, affecting 0.8% of newborns (5). The spectrum of these defects is broad and ranges from mild and hemodynamically insignificant lesions up to severe and complex conditions sometimes needing multiple interventions during childhood, such as functionally univentricular hearts. The incidence of different heart defects varies, with ventricular septum defects apparently the most common type of CHD. See Table 1 for an overview of types of CHDs and their severity grading.

**TABLE 1:** Severity grading of congenital heart disease (CHD) [modified after Hoffman et al. (5)].

Severe CHD
The majority of these patients present as severely ill in the newborn period or early infancy. Cyanotic heart disease • D-transposition of the great arteries • Tetralogy of Fallot • Hypoplastic right heart: Tricuspid atresia; Pulmonary atresia with intact ventricular septum; Ebstein anomaly • Hypoplastic left heart: Aortic atresia; Mitral atresia • Single ventricle • Double outlet right ventricle (DORV) • Truncus arteriosus communis (TAC) • Total anomalous pulmonary venous connection (TAPVC) • Critical pulmonary stenosis (PS) • Miscellaneous uncommon lesions Acyanotic lesions • Atrioventricular septal defect (AVSD) • Large ventricular septal defect (VSD) • Large persistant ductus arteriosus Botalli (PDA) • Critical or severe aortic stenosis (AS) • Severe pulmonary stenosis (PS) • Critical Coarctation (CoA)
Moderate CHD
These patients require expert care, but less intensive than those with severe CHD. Most conditions are detected during childhood. • Mild or moderate aortic stenosis or regurgitation (AS/AR) • Moderate pulmonary stenosis or regurgitation (PS/PR) • Non-critical CoA • Large atrial septal defect (ASD) • Complex forms of VSD
Mild CHD
This is the most numerous group and patients are asymptomatic. They often undergo spontaneous resolution of their lesions. • Small VSD • Small PDA • Mild PS • Bicuspid aortic valve without AS or AR • Small or spontaneously closed ASD

Historically, most patients with severe CHD died in early childhood. However, over the past decades, the life expectancy of these children has increased significantly due to the extraordinary advances in the field of congenital heart surgery, interventional pediatric cardiology and pediatric intensive care. Current data demonstrates that approximately 90% of infants born with CHD now reach adulthood (6–8). But even after successful treatment, the burden of disease can be significant for these children, as well as their families, and particularly siblings (9).

A majority of children with AHD and palliative care needs have single-ventricle physiology (approximately half of them with hypoplastic left heart syndrome), followed by Tetralogy of Fallot with pulmonary artresia, double outlet right ventricle, complete atrioventricular canal type, pulmonary vein stenosis and other severe valve diseases. In addition to congenital, mostly structural heart defects, some children suffer from cardiomyopathy, pulmonary hypertension, myocarditis or complications from heart transplantation. In many children (50–70%) the diagnosis is made prenatally (10–13).

In all of these children medical care remains highly complex up to death (2, 3, 11, 13–15). This includes a wide range of interventions and technical support, such as mechanical ventilation, extracorporeal membrane oxygenation (ECMO), a ventricular assist device (VAD), tracheostomy, gastrostomy tubes, and peritoneal drains. Similar findings are reported in the PELICAN (Pediatric End-of Life-Care Needs in Switzerland) study, a nationwide retrospective study in Switzerland that analyses data on the last 4 weeks of life in children (0–18 years) who died in the years 2011 or 2012 due to cardiac, neurological or oncological conditions, or during the neonatal period (11, 16). Children with a cardiac condition (19 of the 149 included patients) had a median age of 0.5 years (0.1–9.1 years), were predominantly hospitalized in an ICU (67%), and underwent several interventions (interventions requiring anesthesia 11 of 19; mechanical ventilation 14 of 19; ECMO 4 of 19, tube feeding 17 of 19) and received a high range of medications during the last week of life (3–46, including pain medication in 18 of 19 cases).

Most children with AHD die in ICU settings after discontinuation of life-sustaining interventions (2, 3, 11, 14, 15). Prior to death these children often experience a considerable amount of suffering (2, 10, 11, 14, 16).

#### Symptoms and Suffering in Children With Advanced Heart Disease

Data on symptom burden in children with AHD is limited. A very recent prospective study with 161 hospitalized patients (54% younger than 2 years) provides an overview of the most common symptoms and associated suffering (17). The most frequent symptoms were pain (68%), fatigue (63%) and breathing difficulties (60%). Parents perceived treatment of symptoms as successful for pain (76%) and breathing difficulties (65%). The least relief was achieved in the context of sleep disturbance (24%), sadness or depression (29%), and fatigue (35%). However, most parents reported that the team had addressed symptoms sufficiently. Patients with low functional status were more likely to experience a high symptom burden and suffering. The authors conclude that children with AHD may have an even greater risk of psychiatric morbidity, particularly anxiety and/or depression than the pediatric oncology population (17). Comparably, parent study participants of the above-mentioned PELICAN study reported pain and breathing problems most frequently. When asked to rank the three symptoms that the child suffered from most and were most stressful, parents frequently placed agitation and anxiety in first or second place (16).

An earlier cross-sectional survey that included bereaved parents from two tertiary centers found parents mentioning the poor or fair quality of life (QOL) of their child during its last month of life (14). This is also confirmed by a large study with 475 parents and their 347 children living with mild to complex or severe cardiovascular disease without PPC services being involved (18). Based on self-report, the mean scores of the Pediatric Quality of Life Inventory (PedsQL), which included a cardiac module, were significantly lower than in healthy child norms.

#### Prognostication and Advance Care Planning

Prognostication in children with AHD seems to be even more challenging than principally assumed (13, 14, 19–21). Particularly in pediatrics, prognostication should not be reduced to questions of “how long” or the binary “curable/incurable,” but include a comprehensive conversation about patients’ and families’ expectations, needs and hopes, their understanding of disease and prognosis and the QOL of the affected child and the whole family (19). Many parents do not realize and struggle to accept that their child has no realistic chance for survival and do not feel prepared for their child’s dying and medical problems which may arise prior to death (10, 14).

A longitudinal survey by Morell et al. (13) showed the importance of prognostic understanding in parents, as this was associated with greater preparedness for the child’s medical problems. The perspectives of parents and physicians differed significantly concerning prognosis and burden of disease. In particular, parents of children who had had cardiac surgery during the survey reported a significantly poorer understanding of prognosis. This was interpreted as being a result of the day-to-day management of the child’s disease in hospital. Nevertheless and as is also well known in pediatric oncology (22, 23), parents of children with complex heart disease wish to receive more information about the disease in general and the child’s prognosis, even if this includes “bad news” (13, 16).

In specialties such as cardiology or oncology, where medical and surgical advances have led to tremendous change, parallel planning should be accorded growing importance. This means the introduction of PPC and ACP alongside disease-directed, cure-seeking, life-prolonging treatment and interventions as long as they are in the child’s best interest (24).

#### Early Involvement of Pediatric Palliative Care for Cardiac Patients

Early involvement of PPC could enhance support for patients and their families throughout the course of disease (25, 26). This may also help to move away from the misconception of PPC as EOL care only. Contact with the PPC team may allow more space for hope, which can be important for coping with the most complex situations and promoting a sense of security and trust (27, 28). If there is space for hope, PPC involvement may also provide a space to reflect on feelings of doubt about continuing a burdensome and failing treatment (25).

The aforementioned recent study from Boston (2) listed the following indications for sPPC referral according to their frequency: goals of care and ACP (76%), longitudinal support (70%), symptom management (37%), complex decision-making (34%) and hospice referral or care coordination (3%). In the later period of analysis (2015–2018), sPPC referral occurred earlier, at 69 days instead of the 21 days prior to death reported for the earlier period. Involvement of sPPC was associated with less invasive treatments, such as mechanical ventilation, inotrope treatment, ECMO or VAD, but higher rates of ACP meetings and documentation of resuscitation status. In addition, sPPC had an influence on hospital charges on the day of death and for the 7 days before, which were significantly lower.

Hancock and his team at the University of Michigan carried out a randomized controlled trial that included mothers of infants with prenatal diagnoses of single-ventricle heart disease to study depression, anxiety, coping and QOL at a prenatal visit (baseline assessment) and at neonatal discharge (29). Mothers were randomized to receive early sPPC, including structured evaluation, psychosocial/spiritual and communication support when the infant was admitted for surgery versus standard care. In the cohort of 38 mothers and neonates, 18 received sPPC and 20 standard care. Mothers with early sPPC self-reported better adaptive coping mechanisms, less maternal anxiety, and improved family relationships.

#### Barriers for the Involvement of Palliative Care

Although pediatric cardiologists and cardiac surgeons feel PPC consultations are helpful, many feel the timing is too late and at the same time they perceive resistance concerning the involvement of PPC (21). Barriers to PPC are mainly related to the concern of the unintended message given by introducing PPC: “we are giving up on the child” (21). Another barrier to PPC may be due to the unpredictability of these generally rare conditions, which may discourage the discussion of goals of care (26).

## Discussion

This mini review leads to the following five salient topics that may be of importance to adult and pediatric cardiologists alike:

(1) Despite tremendous successes in pediatric heart surgery, interventional pediatric cardiology and pediatric intensive care, children with AHD carry a high risk of long-term sequelae, suffering and premature death.
(2) In a palliative phase of the disease and at EOL, the symptom burden of these children is high and often underestimated.
(3) Many parents are not prepared for their child’s medical problems and, as a consequence, for their potential death as well as their actual dying.
(4) Prognostic understanding in parents lags behind physicians’ understanding.
(5) For these severely ill children and their families sPPC has been shown to provide benefit in terms of support, improvement of QOL and potentially less invasive treatment prior to death.

At a first glance, these five topics contain contradictory information. However, they reflect an important facet of modern medicine: the downsides of success. Success carries the risk of distracting attention from the consequences of that success. This may explain why many parents of children with AHD do not feel prepared for their child’s medical problems (13) and, as a consequence also feel unprepared for their child’s death (10), even if death is rarely unexpected in these children and adults.

Communication plays a central role in this context as it does in medical care generally. Transparency could help to prevent parents and/or patients from not being or not feeling informed, from missing out on important information or observations, from misconceptions of treatment goals or approaches such as palliative care, and probably from unnecessary surprises. Thus, we need to ask ourselves how it can happen that physicians underestimate symptom-burden, as perceived by parents (13). The same may be true concerning health-related QOL. Furthermore, this extends to the issue of parents not feeling prepared for their child’s medical problems, including poor prognosis (13), and dying (10). Morell et al. (13) write: “… if we can improve parent understanding of prognosis, we can improve how prepared parents feel for the medical problems their child is facing.” One step in this direction might be a conversation anticipating and addressing uncertainty with the help of “what ifs” (30). This allows parents to express their worries and fears and may help to also initiate ACP. The roadmap for a “what if” conversation developed by Snaman et al. (30) could be a very helpful basis. Besides providing explicit information, the parent-physician relationship appears vital to improving parent understanding and avoiding overly optimistic parental expectations (13, 14, 31).

How could this be achieved and what could be the role of sPPC? In children with complex AHD as in other complex chronic and life-limiting conditions, early involvement of sPPC could help to meet the challenges of many different tasks and requirements and provide continuity and coordination of care. This would also allow conversations about prognosis and, if needed, ACP in times of stability, ideally before scheduled interventions or decompensations (13). As teams for children with complex AHD are naturally large, particularly during hospitalization, including various disciplines from cardiology to heart surgery and intensive care and numerous professions from physicians to nurses, physiotherapists, psychologists, social workers, nutritionists, kindergarten teachers, chaplains and others, continuity and coordination of care reaches its limits. Besides the most urgent medical and nursing requirements, there is a risk that higher-level topics and conversations about a broader perspective will miss out. An sPPC team, not directly involved in these daily, highly complex and specialized medical and nursing requirements could take over tasks of coordination and continuity and facilitate communication, always in close contact with the primary team. The patient’s primary team would maintain the lead and medical responsibility. The sPPC team would be involved in the sense of a consultative service, a model of care that is frequently found internationally, especially in tertiary settings (32). Within the scope of practice of such a sPPC team, new roles can emerge allowing a task-shifting and distribution of responsibilities among the different PPC providers. Trustworthy partnership and structured exchange between teams is a prerequisite for a successful integration of sPPC in the care of these children and their families. The misconception that palliative care is EOL care could thus slowly be dispelled, not only for lay people but also for professionals.

More concretely, patients with AHD and their families could benefit from the early involvement of sPPC in several respects: (1) continuous support of the child and the family; (2) improvement of communication, including regular individual information, if appropriate even for the sick child; (3) improvement of prognostic understanding and timely ACP; (4) early anticipation and comprehensive assessment of symptoms and suffering in the affected child and the family; (5) assessment of rare, hitherto little-considered symptoms such as anxiety and fatigue; (6) awareness and improvement of QOL of the child and the family, and (7) support of the attending teams and healthcare professionals.

### Future Directions and Conclusion

Declaring early integration of sPPC for children with AHD as a goal would need conceptual work at departmental and institutional levels. To further demonstrate the effects of sPPC interventions and to also overcome persistent barriers, it would be helpful to define and evaluate outcomes as has been done in Boston (2) and is ongoing in a multicenter study in Switzerland – SPhAERA (Specialized Pediatric Palliative Care: Assessing family, healthcare professionals and health system outcomes in a multi-site context of various settings) (33).

Specialized pediatric palliative care has the potential to improve the QOL of the affected children as well as their families by relieving suffering on different levels and augmenting ACP. EOL experiences of children with AHD and their families can be influenced through earlier awareness and understanding of a prognosis and probably less invasive therapies at EOL. A close collaboration between teams and healthcare professionals may also positively influence the well-being of highly engaged healthcare professionals.

## Author Contributions

EB developed the concept and structure, and wrote the first draft of the manuscript. SL, KZ, and AO contributed to the writing of the manuscript. All authors reviewed and approved the final manuscript.

## Conflict of Interest

The authors declare that the research was conducted in the absence of any commercial or financial relationships that could be construed as a potential conflict of interest.

## Publisher’s Note

All claims expressed in this article are solely those of the authors and do not necessarily represent those of their affiliated organizations, or those of the publisher, the editors and the reviewers. Any product that may be evaluated in this article, or claim that may be made by its manufacturer, is not guaranteed or endorsed by the publisher.
